# Reverse transcriptase drug resistance mutations in HIV-1 subtype C infected patients on ART in Karonga District, Malawi

**DOI:** 10.1186/1742-6405-8-38

**Published:** 2011-10-13

**Authors:** Vijay B Bansode, Simon AA Travers, Amelia C Crampin, Bagrey Ngwira, Neil French, Judith R Glynn, Grace P McCormack

**Affiliations:** 1 Molecular Evolution and Systematics Laboratory, Zoology, Ryan Institute, School of Natural Sciences, National University of Ireland, Galway, Ireland; 2 South African National Bioinformatics Institute, University of the Western Cape, Bellville, South Africa; 3 Karonga Prevention Study, Chilumba, Malawi; 4 Department of Epidemiology and Population Health, London School of Hygiene and Tropical Medicine, London, UK

**Keywords:** HIV-1, drug resistance, subtype C, ART, Malawi, Reverse transcriptase

## Abstract

**Background:**

Drug resistance testing before initiation of, or during, antiretroviral therapy (ART) is not routinely performed in resource-limited settings. High levels of viral resistance circulating within the population will have impact on treatment programs by increasing the chances of transmission of resistant strains and treatment failure. Here, we investigate Drug Resistance Mutations (DRMs) from blood samples obtained at regular intervals from patients on ART (Baseline-22 months) in Karonga District, Malawi. One hundred and forty nine reverse transcriptase (RT) consensus sequences were obtained via nested PCR and automated sequencing from blood samples collected at three-month intervals from 75 HIV-1 subtype C infected individuals in the ART programme.

**Results:**

Fifteen individuals showed DRMs, and in ten individuals DRMs were seen from baseline samples (reported to be ART naïve). Three individuals in whom no DRMs were observed at baseline showed the emergence of DRMs during ART exposure. Four individuals who did show DRMs at baseline showed additional DRMs at subsequent time points, while two individuals showed evidence of DRMs at baseline and either no DRMs, or different DRMs, at later timepoints. Three individuals had immune failure but none appeared to be failing clinically.

**Conclusion:**

Despite the presence of DRMs to drugs included in the current regimen in some individuals, and immune failure in three, no signs of clinical failure were seen during this study. This cohort will continue to be monitored as part of the Karonga Prevention Study so that the long-term impact of these mutations can be assessed. Documenting proviral population is also important in monitoring the emergence of drug resistance as selective pressure provided by ART compromises the current plasma population, archived viruses can re-emerge

## Introduction

It has been estimated that in sub-Saharan Africa, approximately 3.9 million people have started antiretroviral treatment (ART) since its introduction (UNAIDS, 2010). Given the large population on treatment, viral diversity coupled with low adherence could lead to the emergence and large-scale transmission of drug resistant strains. Rates of drug resistance among patients who received ART in sub-Saharan Africa range from 3.7%-49% after 24-163 weeks of HAART [1]. Various factors contribute to this large range in resistance among African cohorts such as variation in available healthcare systems and practices, adherence, and access to monitoring [2]. Development of DRMs to Trioimmune^®^, the drug combination used as first line therapy in Karonga District, Malawi, has been reported in Zambia [3], South Africa [4], Cameroon [5], Kenya [6] and Uganda [7]. Previous studies on drug resistance in Malawi showed various DRMs to both NRTIs and NNRTIs in both drug naïve individuals [8] and those failing therapy [9]. However, very little data is yet available on the emergence of drug resistance to ongoing treatment and the transmission of drug resistant variants in subtype C infected countries [3,5-7,10].

The Malawi antiretroviral treatment (ART) program started in 2004, and between then and the end of June 2010 over 225, 000 patients had initiated first-line antiretroviral therapy (ART) through 396 ART clinics [11]. As part of the Karonga Prevention Study (KPS), investigating how the availability and use of ART may change the HIV epidemic and its socio-demographic impact in the rural Karonga District, (northern Malawi), an ART research cohort was established from those attending the ART clinic at Chilumba Rural Hospital. HIV-1 subtype C is the predominant subtype in this District [12]. The objective of this overall study was to investigate the success of the current ART delivery programme in a rural population, and, as a component of this, to investigate the evolution of drug resistance using a traditional consensus sequence genotyping approach.

## Materials and methods

### Study Participants and Treatment schedules

At the Ministry of Health ART clinic at Chilumba Rural Hospital all those attending for screening for ART suitability and who are resident in a geographically defined area adjacent to the clinic, are invited to take part in an observational cohort study. Every three months participants are clinically assessed by KPS research staff. Blood samples are collected at the their first visit (baseline) and at every follow-up visit. A CD4 count is performed at baseline, 6, 12 and 24 months or at the time of a clinical failure, defined by a new WHO stage 3 or 4 event after six months of therapy (WHO, 2006). First line therapy is a generic fixed-dose combination treatment (Triommune^®^), which consists of: stavudine (d4T), lamivudine (3TC), and nevirapine (NVP). All individuals were on first line therapy only.

### DNA Extraction, PCR and Sequencing

Whole blood samples were collected in 4.5 ml vacutainer tubes. Samples were centrifuged and plasma and cell pellet were stored separately at -70°C. DNA was extracted from whole blood cell pellet samples using the QIAamp DNA Blood Mini Kit (QIAGEN ltd). Extracted DNA was subjected to nested PCR amplification of the HIV-1 reverse transcriptase as described in Bansode et al [13]. All PCR amplicons were gel purified and automatically sequenced.

### Sequence Analyses

Sequence chromatographs were edited in SeqMan (DNASTAR, Inc) and all sites that showed ambiguities (two or more peaks of equal, or almost equal, height) were noted. Multiple alignments were assembled of all subtype C sequences generated with the 57 reverse transcriptase sequences generated from [13] using MacClade 4.0 (Sinauer Assoc). Sequences were submitted for analysis of DRMs to the Stanford Database [14]. To check for transmission of DRMs, phylogenetic trees were reconstructed using the LANL subtype C ancestral sequence as outgroup under the GTR + gamma model of DNA substitution implemented RAxML7.0.3 [15] with all parameters optimised by RAxML. Confidence levels in the groupings in the phylogeny were assessed using 1000 bootstrap replicates as part of the RAxML phylogeny reconstruction.

Permission for the study was received from the National Health Sciences Research Committee, Malawi, and the Ethics Committee of the London School of Hygiene and Tropical Medicine, UK

## Results

One hundred and forty nine subtype C sequences were generated from 75 individuals, 65 of which were from blood samples collected at baseline (and reported to be ART naïve). DRMs were found in sequences from 15 individuals (20%) overall, and for 10 individuals (15.4%) the mutations were found in sequences from baseline samples (drug naïve). Details of observed drug resistance mutations are summarized in Table 1. Seven individuals showed DRMs (or ambiguities that suggest the presence of DRMs) to NRTIs used in Karonga with 6/7 showing the mutation V118I. While ten individuals showed the presence of DRMs to NNRTIs only five showed DRMs against therapies used in Karonga, the most common being Y181C and G190AE.

**Table 1:** Mutations associated with antiretroviral drug resistance found in sequences from HIV-1 subtype C infected individuals from Karonga District Malawi.

Patient	Comments	Sex	Time point (month)	NRTI	NNRTI
Patient 2 *	Immune Failure	F	0	No DRMs	No DRMs
			
			6	No DRMs	**K103KN**
			
			9	No DRMs	**Y181C**
			
			12	No DRMs	No DRMs

Patient 5		F	0	No DRMs	E138A
			
			6	No DRMs	E138A

Patient 12 *	Immune Failure	F	0	No DRMs	No DRMs
			
			3	No DRMs	**Y181NY**
			
			12	No DRMs	E138A

Patient 14		M	12	No DRMs	E138A

Patient 20	Immune Failure	F	0	No DRMs	E138A
			
			3	No DRMs	E138A
			
			6	No DRMs	E138A
			
			9	No DRMs	E138A

Patient 32 *		M	0	No DRMs	V90I
			
			6	**M41MR, T215ST**	No DRMs
			
			12	No DRMs	No DRMs
			
			15	No DRMs	V108AV
			
			22	No DRMs	No DRMs

Patient 42 *		M	0	No DRMs	No DRMs
			
			0	**V118IV**	No DRMs
			
			3	**V118I**	No DRMs

Patient 45		F	0	No DRMs	V106I, E138A, **G190A**
			
			6	No DRMs	V106I, E138A, **G190A**

Patient 61 *		F	0	**V118I**, K219R	No DRMs
			
			9	**V118I**	No DRMs

Patient 66		M	0	No DRMs	No DRMs
			
			9	**V118IV**	No DRMs

Patient 76 *		M	0	No DRMs	V90IV, **Y181CY**, H221HY
			
			3	No DRMs	No DRMs
			
			6	No DRMs	**Y181C**
			
			9	No DRMs	No DRMs

Patient 77		F	0	**V118I**	No DRMs
			
			12	**V118I**	No DRMs

Patient 91			0	**V118I**	No DRMs

Patient 93		M	6	**V118I**	E138A
			
			12	**V118I**	E138A

Patient 95			0	No DRMs	E138A

Mutations in bold are against current ART drugs in use in Karonga District.

* Patients showing discrepancies in DRMs between different timepoints

Some individuals showed a discrepancy in the presence and type of DRMs over time. Three patients (Pt2, Pt12 and Pt66) did not show any DRMs at baseline but showed DRMs at subsequent time points (Table 1). Patient 2 also showed a significant drug resistance-related ambiguity (K103KN) in the consensus sequence at 6 months while a different DRM was seen at 9 months (Y181C). No DRM was seen in the sequence from the 12-month sample. Patient 12 showed a similar pattern, where an NNRTI associated mutation (Y181NY) was present in the sequence collected at 3 months, while the sequences at baseline and 12 months did not show any DRMs (Table 1). Both individuals (patient 2 and patient 12) showed immune failure (their CD4 count did not rise over 200 cells/mm^3 ^after 12 months on ART).

Three patients (Pt32, Pt61 and Pt76) showed DRMs at baseline but different DRMs at later time-points (Table 1) with patient 32 showing a high variation of DRMs across timepoints. The baseline sequence from patient 61 showed V118I and K219R, the latter of which was not found in the sequence at 9 months. In Patient 76, the baseline sequence showed the ambiguity Y181CY, with two additional NNRTI mutations (V90IV and H221HY), the 6 months sequence showed the full DRM at position 181 while sequences retrieved from 3 month and 9 month samples showed no DRMs (Table 1).

There was no evidence of transmission of drug resistant HIV between the individuals examined here. Sequences retrieved from each individual grouped monophyletically in all cases. Few individuals showed their sequences clustering with other patients with high bootstrap support but DRMs were not present in both individuals, e.g. sequences from patient 47 and 77 formed a cluster together and are from the same geographical area but while patient 77 showed DRMs, patient 47 did not (data not shown).

## Discussion

Through genotyping RT from HIV-1 subtype C infected individuals on ART using a consensus sequencing approach, we have shown the presence of mutations associated with drug resistance to the therapy used in Karonga District. Drug resistance to Trioimmune^® ^occurred at an overall rate of 20% of individuals (both drug naïve and drug exposed, which is comparable to rates found in other African countries [4-7,16-18] but, as expected, greater than that described in our previous study (7.5%) [8] which did not include individuals currently on therapy.

Patients 2 and 12, both females, had immune failure prior to ART initiation and continued to exhibit immune failure while on ART (i.e. their CD4 counts did not rise above 200 cells/mm^3 ^after 12 months on therapy). While neither showed DRMs from baseline samples they subsequently showed the DRMs Y181C and Y181NY respectively, which is responsible for high-level resistance to NVP, the NNRTI used in 1^st ^line therapy in Karonga. Patient 2 also showed a DRM (K103KN), after 6^th ^months of ART, which also causes high-level resistance to NVP. For these, and a third individual who also exhibited immune failure, it will be important to monitor the individuals and DRMs at subsequent timepoints in case of continued immune failure and development of clinical failure.

Three drug-naïve individuals (Pt 61, Pt 77, Pt 91) showed V118I while another (Pt 42) showed an ambiguity at this position (V118IV). According to the Stanford HIV drug resistance database, V118I is responsible for low-level resistance to 3TC and possibly to other NRTIs when present with other mutations. The mutation has been reported to occur in ~2% of untreated persons infected with subtype C and with increased frequency in persons receiving multiple NRTIs [19] and so it may not be unexpected to find it in this cohort. It was the only DRM found in all three previous studies of drug resistance in Malawi [9,13,20] and was also reported in subtype C infected drug naïve patients from Zambia [21], Zimbabwe [22] and South Africa [19]. It has been suggested that along with drug resistance, the V118I mutation alone is a marker of advanced HIV infection and disease progression [23]. As no associated mutations were found in the three individuals, and they all had a satisfactory response to treatment, this mutation is probably not significant but may become important if a second mutation were to arise.

Mutation G190A (shown in a female patient 45) according to the Stanford drug resistance database, causes high-level resistance to NVP and intermediate resistance to EFV. The mutation was present at baseline in this individual and could indicate acquisition of drug resistant HIV. However, although all individuals participating in the ART cohort study were reported to be ART naïve, we cannot exclude the possibility that some individuals had received some form of ART previously, (e.g. received prevention of mother to child transmission treatment) and did not disclose this fact. The DRM does not appear to have had any major effect on treatment to date, as this individual also has had a satisfactory response.

Drug resistance mutations were found to emerge in some individuals during ART. Patient 32 showed a number of NNRTI mutations and a number of ambiguities at sites important in susceptibility to NRTIs (e.g. the mutation T215S is one of many transitions between wild type and the mutations Y and F [24]). Most of the ambiguities do not reduce NRTI susceptibility but their presence may suggest that the DRM may also be present [25]. This patient had made additional visits to the clinic outside of the routine ART cohort study because of diabetic complications. Additional sequences produced from samples taken at those additional visits showed further mutations associated with drug resistance to NVP and AZT (M41L, M184I, G190E- data not shown), however he has had a satisfactory response to treatment to date.

This study was based on a consensus sequencing approach from provirus due to the difficulty of amplifying HIV from RNA from individuals on ART. While provirus may not provide as clear a picture of the genotype of the circulating virus as would be retrieved from RNA in individuals who have been infected for long periods of time, it has been shown in patients with virological failure that archived resistance mutations previously detected in the proviral DNA were observed in the sequences obtained from the plasma viruses at the time of virological failure [26]. When the selective pressure provided by ART compromises the current plasma population, archived viruses can re-emerge [27]. Therefore documenting the proviral population is also important in monitoring the emergence of drug resistance. Despite the presence of DRMs to current therapy in some individuals, and immune failure in three, no signs of clinical failure were seen during this study. This cohort will continue to be monitored as part of the Karonga Prevention Study so that the long-term impact of these mutations can be assessed.

## Competing interests

The authors declare that they have no competing interests.

## Authors' contributions

VB carried out the molecular biology work and subsequent analysis; AC, BN, NF and JG participated in design of the study; ST and GM conceived and supervised the study. All authors have read and approved the final manuscript.
